# Group and Individual Mindfulness-Based Cognitive Therapy (MBCT) Are Both Effective: a Pilot Randomized Controlled Trial in Depressed People with a Somatic Disease

**DOI:** 10.1007/s12671-016-0575-z

**Published:** 2016-07-21

**Authors:** Maya J. Schroevers, K. Annika Tovote, Evelien Snippe, Joke Fleer

**Affiliations:** 1 Department of Health Sciences, Health Psychology section, University of Groningen, University Medical Center Groningen, FA 12, Ant Deusinglaan 1, 9713 AV Groningen, The Netherlands; 2 Interdisciplinary Center Psychopathology and Emotion regulation, University of Groningen, University Medical Center Groningen, Groningen, The Netherlands

**Keywords:** Mindfulness-based cognitive therapy, Individual, Depression, Somatic illness, RCT

## Abstract

Depressive symptoms are commonly reported by individuals suffering from a chronic medical condition. Mindfulness-based cognitive therapy (MBCT) has been shown to be an effective psychological intervention for reducing depressive symptoms in a range of populations. MBCT is traditionally given in a group format. The aim of the current pilot RCT was to examine the effects of group-based MBCT and individually based MBCT for reducing depressive symptoms in adults suffering from one or more somatic diseases. In this study, 56 people with a somatic condition and comorbid depressive symptoms (i.e., Beck Depression Inventory-II [BDI-II] ≥14) were randomized to group MBCT (*n* = 28) or individual MBCT (*n* = 28). Patients filled out questionnaires at three points in time (i.e., pre-intervention, post-intervention, 3 months follow-up). Primary outcome measure was severity of depressive symptoms. Anxiety and positive well-being as well as mindfulness and self-compassion were also assessed. We found significant improvements in all outcomes in those receiving group or individual MBCT, with no significant differences between the two conditions regarding these improvements. Although preliminary (given the pilot nature and lack of control group), results suggest that both group MBCT and individual MBCT are associated with improvements in psychological well-being and enhanced skills of mindfulness and self-compassion in individuals with a chronic somatic condition and comorbid depressive symptoms. Our findings merit future non-inferiority trials in larger samples to be able to draw more firm conclusions about the effectiveness of both formats of MBCT.

## Introduction

The presence of a chronic somatic disease plays a prominent role in the development of depression, with about 9−23 % of people with one or more chronic somatic diseases having comorbid depression (Moussavi et al. 2007). Given this high prevalence, the burden of depression, and its negative consequences for self-care and medical treatment adherence, it is crucial to develop and test treatments that are acceptable and effective in reducing depressive symptoms. In terms of treatment preferences, the majority of persons experiencing depressive symptoms prefer psychological rather than antidepressant medication (Dwight-Johnson et al. 2000).

Mindfulness-based cognitive therapy (MBCT) is one of the available psychological interventions that focus on reducing depressive symptoms (Segal et al. 2002). Mindfulness refers to being aware of the present moment, by intentionally paying attention without judgment (Kabat-Zinn 2003). MBCT is based on mindfulness-based stress reduction (MBSR), developed by Kabat-Zinn (1979), a group program helping people to cope with severe medical conditions and their psychological impact. MBCT combines mindfulness exercises such as meditation with cognitive behavioral exercises and psycho-education about depression. MBCT is a structured 8-week group program, with weekly 2- to 2.5-h sessions. Doing daily homework exercises is a central part of MBCT. MBCT was specifically developed to teach formerly depressed people to prevent relapse (Teasdale et al. 2000), but is currently also employed for treating current depressive symptoms. Meta-analyses concluded that mindfulness-based interventions are effective for relapse prevention (Piet and Hougaard 2011; Fjorback et al. 2011) as well as reducing current depressive symptoms in both healthy populations (Chiesa and Serretti 2009), clinical populations (Hofmann et al. 2010), and somatic populations (Piet et al. 2012), with effect sizes being comparable to those of alternative type of treatments, including cognitive behavioral therapy (CBT) (Beltman et al. 2010).

Mindfulness-based interventions are usually given in groups of 8 to 12 participants, and most research on the efficacy of mindfulness-based interventions is based on these group interventions. So far, very few studies have examined whether this group format of MBCT is more effective than alternative formats, particularly an individual delivery. Both clearly have advantages and disadvantages. The group delivery of MBCT may provide participants observational learning, encouragement, emotional support, a sense of common humanity and a wider perspective on problems, and possibly an increased motivation to do daily homework exercises. In addition, group sessions cost much less than individual sessions. Yet, this group format may not be the right format for everyone. Very little is known about this. One study in employees from large healthcare organizations on preferences for MBCT suggests that a subgroup of people preferred individual MBCT rather than group MBCT (Lau et al. 2012). Another recent study also suggest that people may prefer individual over group format (Wahbeh et al. 2014b). A qualitative study in cardiac patients participating in an MBCT course reported that although many patients found the group experience to be normalizing and supporting (Griffiths et al. 2009), some patients found the group sharing frustrating and disappointing.

An individual format may particularly be beneficial for people who otherwise do not want to participate in a mindfulness-based intervention or are not able to do so, due to severe illness, disabilities or disabling symptoms like pain or fatigue, or constrained time schedules. When offering MBCT individually, the trainer can adapt the setting and timing to the specific needs of the patient and give full attention to the person. Another advantage could be that participants are not overwhelmed by the stories and difficulties of others. Yet, disadvantages are the lack of encouragement, a sense of common humanity, and motivation to do daily homework exercises by other participants. On the other hand, this format may be more feasible, as it might also not always be possible to participate or offer a group program. This may explain why trainers in clinical practice have started to offer MBCT in an individual format, even while there is currently a lack of empirical evidence for the efficacy of this format. In addition, this individual format is likely to be much more costly compared to the group delivery of the mindfulness-based program. One uncontrolled, small pilot study in persons with elevated stress, examining a 6-weekly 90-min individual MBCT program, found promising results, with significant decreases in negative affect and increases in mindfulness (Wahbeh et al. 2014a).

Given the lack of empirical evidence for the efficacy of an individual format of MBCT, more research is needed about the possible benefits of this type of MBCT. Recently, a structured individual MBCT protocol was developed, adapting the standardized group MBCT into an 8-week individual program, hereby following the structure and content of each session and the type of homework as much as possible (Schroevers et al. 2015). In a pilot RCT study in depressed patients with diabetes, individual MBCT was found to be effective in reducing depressive symptoms, compared to a waitlist control condition (Schroevers et al. 2015). In a larger trial, also in depressed patients with diabetes, the efficacy of individually administered MBCT and individually administered CBT was examined in comparison to a waiting list control condition, showing that both interventions were equally effective in reducing depressive symptoms (Tovote et al. 2014).

The aim of the present pilot RCT study was to examine the effects of individual MBCT and group MBCT on depressive symptoms in depressed people with one or more chronic somatic diseases. We also examined effects on secondary outcomes, including anxiety, positive well-being, mindfulness, and self-compassion. Although set up as a pilot and not as a non-inferiority trial, we also explored differences between group MBCT and individual MBCT in their effects. Patients filled out questionnaires at three points in time (i.e., pre-intervention, post-intervention, 3 months follow-up). We expected both formats of MBCT to be equally effective, with medium effect sizes.

## Method

### Participants

Eligible participants were patients with a chronic somatic disease, as diagnosed by their general practitioner (GP), aged ≥18 and ≤70 years, with depressive symptoms in the past 2 weeks as indicated by a Beck Depression Inventory-II (BDI-II) score ≥14 (cutoff score indicating the presence of at least mild symptoms of depression). Exclusion criteria were as follows: not being able to read and write Dutch, the presence of severe psychiatric comorbidity or cognitive impairment, acute suicidal ideations or behavior, receiving an alternative psychological treatment during or less than 2 months prior to study inclusion, and new or unstable treatment with an antidepressant in the last 2 months prior to study inclusion.

### Procedure

This study was a pilot randomized controlled trial with two active treatment conditions, namely, group MBCT and individual MBCT. The research protocol was reviewed and accepted by the Medical Ethical Committee of the University Medical Center Groningen (UMCG), the Netherlands. The study was conducted in accordance with the principles of the Declaration of Helsinki (version 2008) and the Medical Research Involving Human Subjects Act (WMO).

**Fig. 1:**
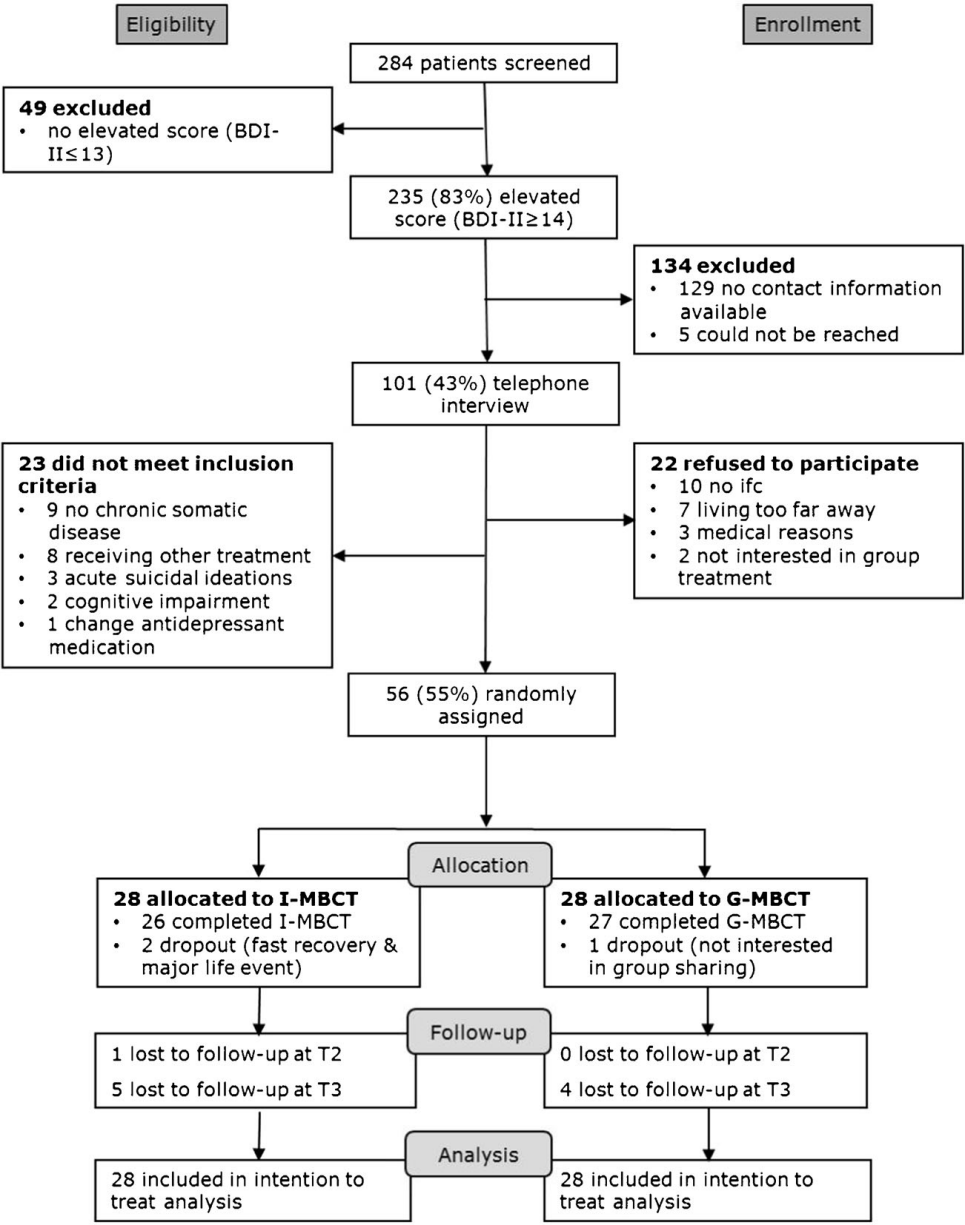
Flowchart of study inclusion

Patients were recruited from October to December 2013. We used a convenience sample, recruited by advertisements in local newspapers. In the advertisement, people were informed that the aim of the study was to examine the effects of a mindfulness-based intervention on mood and that the study would use randomization to allocate people to one of two formats. Those interested in study participation were asked to fill in a screening questionnaire (i.e., BDI-II, see measures) (Fig. 1). When reporting elevated levels of depressive symptoms, patients were called for a telephone interview to check all eligibility criteria. Eligible patients providing written informed consent were randomized into group or individual MBCT. Computerized randomization was carried out stratified by gender, baseline BDI-II, and use of antidepressant medication. Allocation was concealed, as the researcher admitting persons to the study was not aware of the upcoming assignment by the computerized randomization program. Two thirds of the randomized participants received MBCT (group or individual) in October–December; the other third received the training in January and February in 2014.

Both group and individual MBCTs are structured interventions aimed at reducing depressive symptoms, and the MBCT trainers used a detailed session-by-session protocol. The group MBCT intervention was based on the standardized and well-described 8-week MBCT group protocol (Segal et al. 2002). The individual MBCT intervention was an adapted version of the standardized 8-week MBCT group protocol. We have written an extensive treatment manual, including a detailed session-by-session description, transcripts of all exercises, and descriptions of how to perform the enquiry (for details, see Tovote et al. 2014). In short, the 8-weekly group sessions of 120–150 min were adapted to 8-weekly individual sessions of 60 min; as a consequence, we shortened the duration of the exercises within the sessions and took out the cognitive exercise during the second session. For both group and individual MBCTs, participants received an informational booklet and CDs with guided exercises during the first session. CDs were based on the Dutch version of the guided exercises that accompanied Segal et al. (2002), lasting 30 min each. Participants in both conditions were asked to practice at home every day for at least 30 min. Homework assignments consisted of the guided mindfulness exercises on the CD together with informal exercises such as mindful eating. Both interventions were delivered by trained therapists who received a 3-day training by an experienced, qualified mindfulness trainer and supervision every 3 weeks throughout the intervention period.

### Measures

Patients filled out self-report questionnaires at their homes (i.e., without the trainer or researcher being present) at three points in time (i.e., pre-intervention, post-intervention, 3 months follow-up), including scales to assess depressive symptoms, anxiety, positive well-being, mindfulness, and self-compassion (see below).

Data on demographic characteristics (i.e., age, gender, education, employment, and marital status) and the presence of chronic somatic diseases were collected at pre-intervention. At post-intervention, we asked people about their preference for individual or group MBCT at the start of the study (on a five-point scale, ranging from a strong preference for group MBCT to a strong preference for individual MBCT) and their satisfaction with the actually received format of MBCT (on a five-point scale, ranging from very satisfied to very dissatisfied).

The primary outcome measure, severity of depressive symptoms, was assessed with the BDI-II (Beck et al. 1996). The BDI-II is a 21-item self-report questionnaire, measuring symptoms of depression such as sadness, loss of interest, and hopelessness during the last 2 weeks. The presence of each symptom is rated on a four-point scale ranging from 0 (“not at all”) to 3 (“most of the time”). A score from 14 to 19 indicates mild depression, a score from 20 to 28 indicates moderate depression, and a score ≥29 indicates severe depression. The reliability of the BDI-II as indicated by Cronbach’s alpha was adequate in the current study (*α* = 0.77–0.95, for the different time points).

Anxiety was assessed by the Generalized Anxiety Disorder 7 (GAD-7), a seven-item self-report instrument (Spitzer et al. 2006). Respondents are asked to report the frequency with which they experience worrying, feeling restless, annoyed, or afraid during the past 2 weeks. Each item is scored 0 (“not at all”) to 3 (“nearly every day”). A total sum score of 5 to 10 indicates mild anxiety, a score of 11 to 15 indicates moderate anxiety, and a score of >15 indicates severe anxiety. The reliability in this study was good (*α* = 0.79–0.90, for the different time points).

Positive well-being was assessed with the Well-Being Index (WHO-5) (Bech et al. 2003). This self-report instrument consists of five items that are scored on a six-point scale from 0 (“not present”) to 5 (“constantly present”). The items are about positive mood, vitality, and general interest in relation to the last 2 weeks. The total sum score is converted to a score between 0 and 100, with a score ≤50 indicating poor well-being. In this study, the scale’s reliability was good (*α* = 0.83–0.92, for the different time points).

Mindfulness was assessed with the Five Facet Mindfulness Questionnaire (FFMQ), which is one of the most widely used mindfulness questionnaires (Baer et al. 2006). This 39-item scale assesses five components of mindfulness: observing, describing, non-judging of experience, acting with awareness, and non-reactivity to inner experience. Participants rated the degree to which every statement is true on a five-point Likert scale ranging from 1 (“never or very rarely true”) to 5 (“very often or always true”). A total score was calculated with higher scores indicating greater levels of mindfulness. In this study, the reliability was good (*α* = 0.85–0.94, for the different time points).

Self-compassion was assessed with the Self-Compassion Scale—Short Form (Raes et al. 2011), which has a nearly perfect correlation with the long scale when examining total scores (Neff 2003). The scale consists of 12 items that can be answered on a five-point Likert scale, ranging from 1, “seldom or never,” to 5, “(almost) always”. It measures components of self-compassion like self-kindness, common humanity, self-judgement, isolation, over-identification, and mindfulness. The short form demonstrated adequate reliability (Raes et al. 2011). Also in this study, the reliability was good (*α* = 0.85–0.89, for the different time points).

### Data Analyses

SPSS Statistics 22 was used for all analyses. Data were analyzed by the second author (not blinded). To investigate if there were differences at baseline between the groups regarding demographic and clinical variables as well as primary and secondary outcome measures, *t* tests, chi-squared tests, and Fisher’s exact tests were used. Intention-to-treat methods were used for all analyses. We performed sensitivity analyses based on participants with no missing data and datasets with different imputation methods (e.g., last observation carried forward, regression imputation, group mean substitution). As all analyses revealed a similar pattern of results, we imputed missing data based on the group mean score and used these scores in the intention-to-treat analyses. In addition to intention to treat, we performed similar analyses on completed data, without imputations. Repeated measures analyses were performed to examine the effects within both intervention conditions. We also tested whether the time × condition (i.e., group versus individual MBCT) was significant, in order to examine differences between the two conditions with respect to treatment effects. Effect sizes were calculated using Cohen’s d (Cohen 1992).

## Results

This study was set up as a pilot study, not as an equivalence trial. Given the resources and time schedule of the project, we had a limited time period to recruit participants for the study. In total, 284 people were screened with the BDI-II, of which 56 people provided informed consent and were randomized to either individual MBCT (*N* = 28) or group MBCT (*N* = 28) (see Fig. 1). As can be seen in Table 1, participating patients were mostly female, on average 52 years old, having a relationship, and highly educated, with no significant differences between group and individual MBCT participants. Two thirds of patients reported having one chronic disease; the other third reported two or more diseases. In total, 77 illnesses were reported by patients, with most common being chronic pain (19 times reported), disorders of joints (16 reported), cardiac disease (8 reported), and diabetes (7 reported). When asked at post-intervention, about two thirds of people said that they had preferred individual MBCT at the start of the study (i.e., before randomization). After having received MBCT, people in both conditions were equally satisfied with the actually received format of MBCT.

**Table 1:** Characteristics of the study participants

	Group MBCT	Individual MBCT	Total (*n* = 56)	Differences between
	(*n* = 28)	(*n* = 28)		Group and individual MBCT
Age (years) *M (SD)*	52.1 (9.9)	52.4 (11.7)	52.2 (10.7)	*t* = 0.1
				*p* = 0.92
Gender *n (%)*				
Male	7 (25 %)	6 (21 %)	13 (23 %)	*χ* ^2^ = 0.10
Female	21 (75 %)	22 (79 %)	43 (77 %)	*p* = 0.75
Relationship status *n (%)*				
In a relationship	24 (86 %)	19 (68 %)	43 (77 %)	*χ* ^2^ = 2.50
Not in a relationship	4 (14 %)	9 (32 %)	13 (23 %)	*p* = 0.11
Education *n (%)*				
Lower level vocational school	3 (11 %)	1 (3 %)	4 (7 %)	*p* = 0.35^d^
Secondary education/advanced	13 (46 %)	10 (36 %)	23 (41 %)	
Level vocational school				
Higher or University education	12 (43 %)	17 (61 %)	29 (52 %)	
Employment *n (%)*				
Employed	9 (32 %)	12 (43 %)	21 (38 %)	*χ* ^2^ = 0.69
Not employed	19 (68 %)	16 (57 %)	35 (62 %)	*p* = 0.41
Somatic disease *n (%)*				
One disease	17 (61 %)	20 (71 %)	37 (66 %)	*χ* ^2^ = 0.72
More than one disease	11 (39 %)	8 (29 %)	19 (34 %)	*p* = 0.40
Preference format^a^ *n* (%)^b^				
No preference	6 (21 %)	7 (26 %)	13 (24 %)	*p* = 0.99^d^
Group	5 (18 %)	4 (15 %)	9 (16 %)	
Individual	17 (61 %)	16 (59 %)	33 (60 %)	
Satisfaction received format^c^ (%)^a^				
Satisfied	23 (86 %)	25 (92 %)	48 (90 %)	*p* = 0.70^d^
Neutral	2 (7 %)	1 (4 %)	3 (5 %)	
Dissatisfied	2 (7 %)	1 (4 %)	3 (5 %)	

^a^Patients’ preference for individual or group MBCT at the start of the study was assessed at post-intervention

^b^Data is missing for one person

^c^Satisfaction with the actual received format (i.e., individual or group) was assessed at post-intervention

^d^Fisher’s exact test

Table 2 shows the means and standard deviations on all study variables for both group and individual MBCTs at the three points in time (i.e., imputed missing data). There were no significant pre-intervention differences between the two conditions on any of these variables (*p* > 0.05). Effect sizes refer to Cohen’s d, with values ranging from 0.2 to 0.5 indicating small effects, values from 0.5 to 0.8 indicating moderate effects, and values >0.8 indicating large effects of interventions.

**Table 2:** Means and standard deviations for all study variables in group MBCT and individual MBCT

Measure	Condition	T1 M (SD)	T2 M (SD)	T3 M (SD)	T1–T2 MD (95 % CI)	T1–T3 MD (95 % CI)	Time F	d_T1/T2_	d_T1/T3_
Depression	I-MBCT	25.0 (8.8)	13.1 (10.1)	12.9 (11.0)	11.6 (8.2, 15.5)	12.1 (8.8, 15.4)	41.1*	1.25	1.21
	G-MBCT	24.0 (6.0)	10.8 (5.9)	11.2 (10.0)	13.3 (10.2, 16.4)	12.9 (9.1, 16.6)	37.7*	2.23	1,57
Anxiety	I-MBCT	9.8 (4.3)	6.7 (4.9)	6.7 (4.4)	3.0 (1.7, 4.4)	3.0 (1.7, 4.4)	14.6*	0.66	0.70
	G-MBCT	9.8 (3.7)	5.0 (3.1)	5.6 (4.7)	4.8 (3.3, 6.3)	4.2 (2.1, 6.3)	17.0*	1.40	1.00
Well-being	I-MBCT	28.1 (16.6)	46.1 (20.1)	49.9 (17.7)	−17.9 (−24.9, −11.0)	−21.8 (−29.0, −14.5)	23.1*	0.97	1.27
	G-MBCT	28.9 (15.3)	50.9 (20.9)	53.7 (22.9)	−21.9 (−30.4, −13.5)	−24.8 (−33.4, −16.1)	21.7*	1.20	1.27
Mindfulness	I-MBCT	112.5 (14.5)	129.4 (16.4)	131.0 (18.3)	−16.9 (−23.2, −10.7)	−18.5 (−25.8, −11.2)	23.8*	1.09	1.12
	G-MBCT	114.4 (16.5)	131.2 (14.6)	129.9 (18.1)	−16.8 (−22.1, −11.6)	−15.5 (−20.5, −10.4)	27.3*	1.08	0.90
Self-compassion	I-MBCT	2.5 (0.5)	3.0 (0.6)	3.1 (0.6)	−0.5 (−0.7, −0.2)	−0.6 (−0.8, −0.3)	13.7*	0.85	1.07
	G-MBCT	2.7 (0.6)	3.2 (0.6)	3.3 (0.6)	−0.5 (−0.7, −0.3)	−0.6 (−0.8, −0.5)	26.0*	0.78	0.97

*d*_*T1/T2*_ effect sizes based on within-group changes between T1 and T2, *d*_*T1/T3*_ effect sizes based on within-group changes between T1 and T3. *I-MBCT* individual MBCT, *G-MBCT* group MBCT

*p < 0.001 based on within-group changes between T1 and T3

Using intention-to-treat analyses, we found significant improvements in all outcomes within group MBCT and within individual MBCT (*p* < 0.001), with effect sizes indicating mostly large effects (>0.8) within both conditions. These results indicate that patients in both conditions reported significant reductions in depressive symptoms and anxiety, and increases in well-being, mindfulness, and self-compassion over time. Most change was seen between T1 and T2 and thus between pre- to post-intervention, with improvements maintained till T3, i.e., at 3 months follow-up. We did not find significant two-way time x condition interactions (*p* > 0.20). This indicates that we did not find significant differences between group MBCT and individual MBCT in the improvements in all of our primary and secondary outcomes. In addition, we performed similar analyses, this time using completed data, without imputation. Again, all outcomes showed significant improvements within the two variants of MBCT from T1 to T3 (*p* < 0.001). No interaction effects were found for time x condition (*p* > 0.10).

## Discussion

This is the first RCT that investigated the efficacy of group MBCT and individually delivered MBCT. The study was conducted in patients with one or more chronic somatic condition(s) with comorbid depressive symptoms. It should be noted that it was set up as a pilot study, rather than a non-inferiority trial. As hypothesized, both group and individual MBCTs were associated with significant improvements in depressive symptoms, with no significant difference in improvement between the two conditions. The decrease in depressive symptoms can be regarded as a clinically relevant improvement, as the mean post-intervention levels of depressive symptoms were below the cutoff of the scale (i.e., BDI-II) that we used. Patients in both conditions also reported significant reductions in anxiety and increases in positive well-being, mindfulness, and self-compassion. Again, no significant differences were found between the two conditions with respect to these improvements. We also observed that, post-intervention, most patients were satisfied with the format of MBCT they received. This is important information, as two thirds of people reported that they had preferred individual MBCT at the start of the study.

The positive effects of group MBCT on symptoms of anxiety and depression as well as well-being are in line with the large amount of studies demonstrating the benefits of this group intervention for psychological functioning (Hofmann et al. 2010; Fjorback et al. 2011; Piet et al. 2012). Importantly, we found similar effects in patients receiving individual MBCT. This adds to the evidence from previous RCTs that individual MBCT is effective in reducing symptoms of depression and anxiety and in improving well-being in depressed patients with diabetes (Schroevers et al. 2015; Tovote et al. 2014). Findings are also consistent with results from a recent uncontrolled pilot study on individual mindfulness-based training showing significant decreases in perceived stress and negative affect over time (Wahbeh et al. 2014a).

Our results seem in contrast to findings regarding CBT, with a meta-analysis suggesting that individual CBT is more effective than group CBT for treating depressive symptoms in patients with a somatic disease (Beltman et al. 2010). A meta-analysis on group versus individual therapies for depression found that generally individual therapy was only somewhat more effective than group therapies and this advantage of individual therapies was not significant at follow-up any more (Cuipers et al. 2008). Our results suggest that, with respect to MBCT, both group and individual formats are about equally effective, yet given that our pilot study was not set up as an equivalence trial and is the first to examine this, larger studies are needed to draw more firm conclusions.

It is important to note that both group and individual MBCTs also led to improvements in mindfulness and self-compassion. Similar findings have been reported in previous studies on group MBCT (Kuyken et al. 2010; Nyklicek and Kuijpers 2008). The fact that increases in these skills were also found in the participants receiving individual MBCT suggests that the traditional format for mindfulness-based training in a supportive group environment is not a requirement for cultivating skills to be mindful and self-compassionate. A possible explanation may be that these increases in mindfulness and self-compassion are due to the daily mindfulness exercises that patients do (Snippe et al. 2015a).

Several limitations need to be acknowledged while interpreting the findings. First, as the study was set up as a pilot study, the power (i.e., based on the actual number of patients in the analyses) to detect effects was somewhat reduced. We also did not have a fully powered sample to test equivalence, as this would have required 115 participants in each condition. However, given the relatively small difference in depressive symptoms between the two conditions at T2 and T3, results suggest no meaningful difference in outcomes between the two formats of MBCT. A second limitation is that we had no control group. Therefore, we cannot be certain that the effects are due to MBCT or to a time effect. However, when comparing the reductions in depressive symptoms in this study with a previous study on individual MBCT which did include a control group, it seems likely that the improvements in the current study were strongly due to the interventions. Third, participants were recruited using convenience sampling, which has been associated with selection bias, hereby reducing generalizability of the results. Fourth, we had limited information on patients’ clinical characteristics, with the sample being heterogeneous with respect to the number and type of comorbidity. This limits the generalization of the results to other groups. Fifth, it should be noted that patients’ preference for the format of MBCT (group or individual) was asked retrospectively at the post-intervention assessment, so after having received MBCT. However, it is interesting to see that even while the majority of patients in both formats are then satisfied with the received format, about two thirds did report that, at the start, they preferred individual over group format. Finally, all outcomes were measured by self-report and we had no objectively assessed information available (e.g., clinical interview for assessing depressive symptoms).

To conclude, group MBCT and individual MBCT both seem effective in reducing depressive symptoms in patients with a chronic somatic disease. Given the current use of individual MBCT in clinical practice, without previous empirical evidence to support this, our results are innovative and of clinical importance. In the study, we also observed that participants were generally satisfied with the format of MBCT they received, while beforehand about two thirds had preferred individual MBCT. Previous research has found that patients’ expectations of outcomes of MBCT are predictive of homework compliance, treatment completion, and the amount of improvement in depressive symptoms over the course of MBCT (Snippe et al. 2015b). Given this importance of patients’ expectations, it may be valuable to offer patients the format of MBCT (i.e., group or individual) of which they expect the most positive outcomes, especially given our preliminary evidence that both are equally effective.

Future research, particularly a non-inferiority trial, is needed to confirm our results and to test more firmly whether both group and individual MBCTs are equally effective. Future research is also needed to examine factors that can indicate whether certain subgroups of patients are more likely to benefit from group or individual MBCT or to prefer individual over group MBCT or vice versa. Research in the field of CBT suggests that the underlying mechanisms may also differ between group and individual approaches, with different factors mediating improvements in outcomes (Hedman et al. 2013). More research is needed to examine whether distinct mediating factors explain the effects of group and individual MCBT. This may provide a better insight into the working mechanism of both formats and provide directions on how to optimize their efficacy.
